# Medical Association

**Published:** 1849-05

**Authors:** 


### MEDICAL ASSOCIATION.

This body meets in Boston to-day. Louisville, and, so far as we are informed, Kentucky will be without a representative in it. We are glad to learn, however, that the profession elsewhere appears to be alive to the interest and usefulness of the Association, and that the attendance at this meeting is likely to be general. In our next number we shall be able to report the proceedings of the meeting.

